# Risk Potential for Organ Dysfunction Associated With Sodium Bicarbonate Therapy in Critically Ill Patients With Hemodynamic Worsening

**DOI:** 10.3389/fmed.2021.665907

**Published:** 2021-07-07

**Authors:** Tiehua Wang, Lingxian Yi, Hua Zhang, Tianhao Wang, Jingjing Xi, Lin Zeng, Junlin He, Zhongheng Zhang, Penglin Ma

**Affiliations:** ^1^Critical Care Medicine Department, Peking University Third Hospital, Beijing, China; ^2^Critical Care Medicine Department, Strategic Support Force Characteristic Medical Center of People's Liberation Army, Beijing, China; ^3^Research Center of Clinical Epidemiology, Peking University Third Hospital, Beijing, China; ^4^Emergency Department, The 8th Medical Centre of Chinese People's Liberation Army (PLA) General Hospital, Beijing, China; ^5^Department of Medical Affairs, Shanghai Palan DataRx Co. Ltd., Shanghai, China; ^6^Department of Emergency Medicine, Sir Run Run Shaw Hospital, Zhejiang University School of Medicine, Hangzhou, China; ^7^Critical Care Medicine Department, Guiqian International General Hospital, Guiyang, China

**Keywords:** sodium bicarbonate therapy, metabolic acidosis, critically ill patients, organ dysfunction, hemodynamic status

## Abstract

**Background:** The role of sodium bicarbonate therapy (SBT) remains controversial. This study aimed to investigate whether hemodynamic status before SBT contributed to the heterogeneous outcomes associated with SBT in acute critically ill patients.

**Methods:** We obtained data from patients with metabolic acidosis from the Medical Information Mart for Intensive Care (MIMIC)-III database. Propensity score matching (PSM) was applied to match the SBT group with the control group. Logistic regression and Cox regression were used to analyze a composite of newly “developed or exacerbated organ dysfunction” (d/eOD) within 7 days of ICU admission and 28-day mortality associated with SBT for metabolic acidosis.

**Results:** A total of 1,765 patients with metabolic acidosis were enrolled, and 332 pairs obtained by PSM were applied to the final analyses in the study. An increased incidence of newly d/eOD was observed in the SB group compared with the control group (54.8 vs. 44.6%, *p* < 0.01). Multivariable logistic regression indicated that the adjusted OR of SBT for this composite outcome was no longer significant [OR (95% CI): 1.39 (0.9, 1.85); *p* = 0.164]. This effect of SBT did not change with the quintiles stratified by pH. Interestingly, SBT was associated with an increased risk of the composite of newly d/eOD in the subgroup of patients with worsening hemodynamics before SBT [adjusted OR (95% CI): 3.6 (1.84, 7.22), *p* < 0.001]. Moreover, the risk potential for this composite of outcomes was significantly increased in patients characterized by both worsening [adjusted OR (95% CI): 2.91 (1.54, 5.47), *p* < 0.001] and unchanged hemodynamics [adjusted OR (95% CI): 1.94 (1.01, 3.72), *p* = 0.046] compared to patients with improved hemodynamics before SBT. Our study failed to demonstrate an association between SBT and 28-day mortality in acute critically ill patients with metabolic acidosis.

**Conclusions:** Our findings did not demonstrate an association between SBT and outcomes in critically ill patients with metabolic acidosis. However, patients with either worsening or unchanged hemodynamic status in initial resuscitation had a significantly higher risk potential of newly d/eOD subsequent to SBT.

## Introduction

Acute metabolic acidosis is a frequent and potentially life-threatening condition in patients admitted to intensive care units (ICUs) (1–4). It has been demonstrated that low blood pH impairs cellular bioactivities, which contributes to organ dysfunction (5). As shown in *in vitro* experiments and animal studies (6, 7), severe acidosis resulted in a reduction in the vascular contractile response to catecholamines and decreases in cardiac inotropism and ventricular arrythmia, which led to cardiac dysfunction. Accordingly, intravenous administration of sodium bicarbonate (SB) to reverse severe metabolic acidosis (namely, sodium bicarbonate therapy, SBT) has become common in clinical practice (8). According to previous reports, the proportion of patients receiving SBT ranged from 29 to 67% in a cohort of septic patients with metabolic acidosis (9), a mixed ICU population with metabolic acidosis (1) and cases characterized by severe lactic acidosis (10).

However, the role of sodium bicarbonate therapy (SBT) in restoring metabolic acidosis remained heterogeneous in critically ill patients (11, 12). Findings in Jaber's randomized controlled trial (RCT) and Zhang's retrospective cohort study suggested that SBT conferred a potential benefit for outcomes in a highly selected subgroup of patients with acute kidney injury (AKI) (9, 13). Furthermore, the pooled effect of SBT on either hemodynamic stability or mortality was inconclusive among critically ill patients with metabolic acidosis, although increased blood pH or excess alkalinity was evident (14–16). On the other hand, SBT may be harmful owing to its potential induction of hypokalemia, hypernatremia, hypocalcemia, prolonged QTC interval, progression of vascular calcification, rebound metabolic alkalosis, and increased lactate production (17, 18). Given these concerns, there are warnings associated with the use of SBT for the management of critically ill patients in the currently updated multidisciplinary guidelines (19–21).

It is well-known that metabolic acidosis is largely attributable to poor tissue perfusion in the acute phase of critical illness, which plays an important role in the development of organ dysfunction (22). Restoration of hemodynamics as early as possible has been strongly recommended for the initial management of critically ill patients in evidence-based guidelines, although the etiology of hypoperfusion is complex (19, 20). Therefore, SBT should not be considered until hemodynamic optimization has been completed for these patients. However, there have been few previous studies reporting hemodynamic status before the administration of SB (9, 13–15).

Despite multiple factors, we hypothesized that hemodynamic status before SBT could be an important determinant of the heterogeneity of outcomes associated with SBT in acute critically ill patients. Based on the Medical Information Mart for Intensive Care (MIMIC-III) database, this study tested this hypothesis in a cohort of patients who were diagnosed with metabolic acidosis within 48 h of ICU admission.

## Materials and Methods

We obtained data from the MIMIC-III, which is a large US-based, open-access, deidentified critical care database. The newest version of MIMIC-III, v. 1.4, integrates 61,532 patients admitted to the ICUs of Beth Israel Deaconess Medical Center in Boston, Massachusetts, including 53,432 distinct hospital admissions for adult patients aged 16 years or above. The duration of the database covers from June 2001 to October 2012. Since our study was based on a third party anonymized publicly available database with pre-existing institutional review board (IRB) approval, IRB approval from our institution was exempted.

### Study Population

Our study included patients with severe metabolic acidosis within 48 h after ICU admission who fulfilled all of the following criteria: (1) pH ≤ 7.3; (2) BE ≤ −8, or ${\text{HCO}}_{3}^{-}$ < 20 mmol/L if BE was missing; and (3) PaCO_2_ < 50 mmHg, or without chronic obstructive pulmonary disease (COPD) in discharge diagnoses if PaCO_2_ was unavailable. The lowest values of pH, BE, and ${\text{HCO}}_{3}^{-}$ and the highest value of PaCO_2_ were selected when there were multiple measurements. The exclusion criteria included (1) age 16 or under; (2) diabetes insipidus; (3) comorbidity of peripheral paralysis; (4) ICU stay over 100 days; (5) SBT was initiated before ICU admission, later than 48 h after ICU admission or after a record of order for renal replacement therapy (RRT); and (6) death within the first 24 h after ICU admission. For those who had multiple admissions to the ICU, only the initial visit was included.

### Definitions

We defined a composite of newly “developed or exacerbated organ dysfunction” (d/eOD) as a net increase in Sequential Organ Failure Assessment (SOFA) score ≥ 2 of at least one organ within 7 days of ICU admission over the baseline (SOFA score in the first 24 h). The worst SOFA score was adopted if multiple measurements were documented from the second 24-h period (or the day following SBT) until day 7 after ICU admission. Hemodynamic status during the initial resuscitation was defined as hemodynamic improvement, hemodynamic worsening and unchanged hemodynamics. The period of initial resuscitation was defined as the first 6 h of ICU admission. However, it was modified to be the period from ICU admission to SB therapy when SB infusion was initiated earlier than 6 h of ICU admission. The status of “hemodynamic improvement” or “hemodynamic worsening” was determined during initial resuscitation (classification in Supplementary Figure 1) if (1) “the reduction from the highest level” or “the increase over the initial dosage” of intravenous continuous infusion of norepinephrine dosage (or identical dosage of other vasopressors, Supplementary Table 1) was equal to or over 0.1 μg/min/kg (or ≥ 50% net change at least when the highest or initial dosage was below 0.1 μg/min/kg) for maintaining mean arterial pressure (MAP) ≥65 mmHg; or (2) the plasma lactate concentration was “decreased below” or “raised over” 2 mmol/L (or ≥10% net change) while vasopressor data were unavailable. If the change in vasopressors or plasma concentration of lactate did not fulfill either the hemodynamic improvement or hemodynamic worsening criteria, a status of unchanged hemodynamics during initial resuscitation was determined. Sepsis 3.0 definitions were used to categorize the enrolled patients as non-sepsis, sepsis or septic shock (23). AKI stage was identified by the Kidney Disease: Improving Global Outcomes (KDIGO) classification system (24).

### Variables

Characteristic data of patients were extracted, including age and sex; category of diseases (surgical vs. medical), history of comorbidity and whether cardiopulmonary resuscitation (CPR) was experienced; and diagnosis of sepsis, shock and AKI (Table 1). Supportive interventions such as mechanical ventilation (MV) and RRT were recorded. Fluid balances at 24 and 48 h after ICU admission and the total SB dosage administered within the first 48 h were extracted. The SOFA score including each component was calculated every 24 h if data were available. The Simplified Acute Physiology Score II (SAPSII) was calculated within the first 24 h after ICU admission.

**Table 1:** Characteristics of patients in the SB group and non-SB group after PSM.

	**SB (*N* = 332)**	**Non-SB (*N* = 332)**	***P*-value**
**General characteristics**			
Age (year)	66.5 (54.2, 77.6)	66.2 (52.9, 79.9)	0.705
Sex [male, *n* (%)]	158 (47.6%)	163 (49.1%)	0.756
With comorbidity, *n* (%)	330 (99.4%)	327 (98.5%)	0.451
Category [surgical, *n* (%)]	88 (26.5%)	79 (23.8%)	0.474
Sepsis, *n* (%)	164 (49.4%)	132 (39.8%)	0.016
With shock, *n* (%)	285 (85.8%)	278 (83.7%)	0.517
With AKI, *n* (%)	269 (81.0%)	272 (81.9%)	0.842
Receiving RRT, *n* (%)	37 (11.1%)	40 (12.1%)	0.809
Receiving MV, *n* (%)	219 (66.0%)	179 (53.9%)	0.002
Receiving CPR, *n* (%)	34 (10.2%)	37 (11.1%)	0.802
SAPS II score [M (IQR)]	52 (40, 65)	46 (36, 57)	<0.001
SOFA score [M (IQR)]	8 (6, 12)	6 (4, 9)	<0.001
CFB 24-h [L, M (IQR)]	0.52 (0.28, 0.82)	0.35 (0.12, 0.59)	<0.001
CFB 48-h [L, M(IQR)]	0.69 (0.36, 1.16)	0.46 (0.20, 0.76)	<0.001
SB dosage [mmol, M (IQR)]	60 (30, 150)	0	NA
**Laboratory tests**			
pH	7.21 (7.15, 7.25)	7.22 (7.18, 7.26)	0.012
PaCO_2_ [mmHg, M (IQR)]	37.5 (31, 44)	41 (35, 45)	<0.001
PaO_2_ [mmHg, M (IQR)]	89 (61, 122)	67 (44, 95)	<0.001
AB (mEq/L)	14.5 ± 3.7	14.6 ± 4.4	0.960
BE (mEq/L)	−12.5 (−16, −10)	−11 (−14, −9)	0.001
Lactate (mmol/L)	3.2 (1.7, 6.2)	3.4 (1.8, 5.5)	0.935
Hct (%)	27 (22, 32)	28 (24, 32)	0.162
Hb (g/dL)	9.0 (7.2, 10.7)	9.3 (8.1, 10.5)	0.183

*Baseline characteristics were extracted within 6 h of ICU admission or before SBT if it was initiated earlier. SB, Sodium bicarbonate; SBT, Sodium bicarbonate therapy; PSM, propensity score matching; SOFA, Sequential Organ Failure Assessment; SAPSII, The Simplified Acute Physiology Score II; AB, actual bicarbonate; BE, base excess; Hct, hematocrit; Hb, hemoglobin; AKI, acute kidney injury; RRT, renal replacement therapy; CFB, cumulative fluid balance; MV, mechanical ventilation; CPR, cardiopulmonary resuscitation*.

Laboratory variables included pH, actual bicarbonate (AB), base excess (BE), PaO_2_, PaCO_2_, hematocrit (Hct), hemoglobin (Hb), lactate concentration and electrolytes such as [Na^+^], [K^+^], and [Cl^−^]. We also calculated the anion gap (AG) by the formula AG = [Na^+^] + [K^+^] – [Cl^−^] – [HCO3^−^] (25).

A composite of newly d/eOD within 7 days of ICU admission was used as the primary outcome of this study. The secondary outcome was 28-day mortality.

### Statistical Analysis

Single imputation was applied to fix missing data for variables (with missing data <20%), including pH, BE, PO_2_, PCO_2_, lactate, and the cumulative fluid balance (26). Because only one data point was missing, we applied complete sample analysis for the missing AG data.

Propensity score matching (PSM) was applied to minimize either selection biases or potential confounders for SBT (9). The propensity score was estimated by a multivariate logistic regression model using the following variables: the worst value of pH and BE during initial resuscitation, basic characteristics including age, sex and category of disease, variables with regard to severity of disease such as comorbidity, diagnosis of shock or AKI, and whether MV, CPR and RRT were received. A one-to-one nearest neighbor matching algorithm was applied using a caliper width of 0.02.

Continuous variables are expressed as the mean (standard deviation) or median (upper quantile, lower quantile), and a *t*-test or the Wilcoxon-test was used to compare the differences between groups. Categorical variables are depicted as the number of groups (the number of percentages), and the chi-squared test or Fisher's exact-test was applied.

Univariate logistic regression and Cox regression were used to evaluate the overall effectiveness of SBT without any adjustments. Multivariate logistic regression and Cox regression were applied to evaluate the overall effectiveness of SBT with adjustment for potential confounders suggested by clinical expertise and matching variables that remained imbalanced after PSM with *p* < 0.05. Further, logistic regression and Cox regression were implemented to evaluate the impact of hemodynamic status on SBT-associated outcomes among the SB group. Furthermore, we accounted for the collinearity among variables to be adjusted to select final independent variables. The proportional hazard assumption for each independent variable was evaluated based on Schoenfeld residuals.

All statistical analyses were performed by R (version 3.6.1). A *p*-value < 0.05 was indicative of statistical significance.

## Results

### Characteristics of the Enrolled Patients

**Figure 1:**
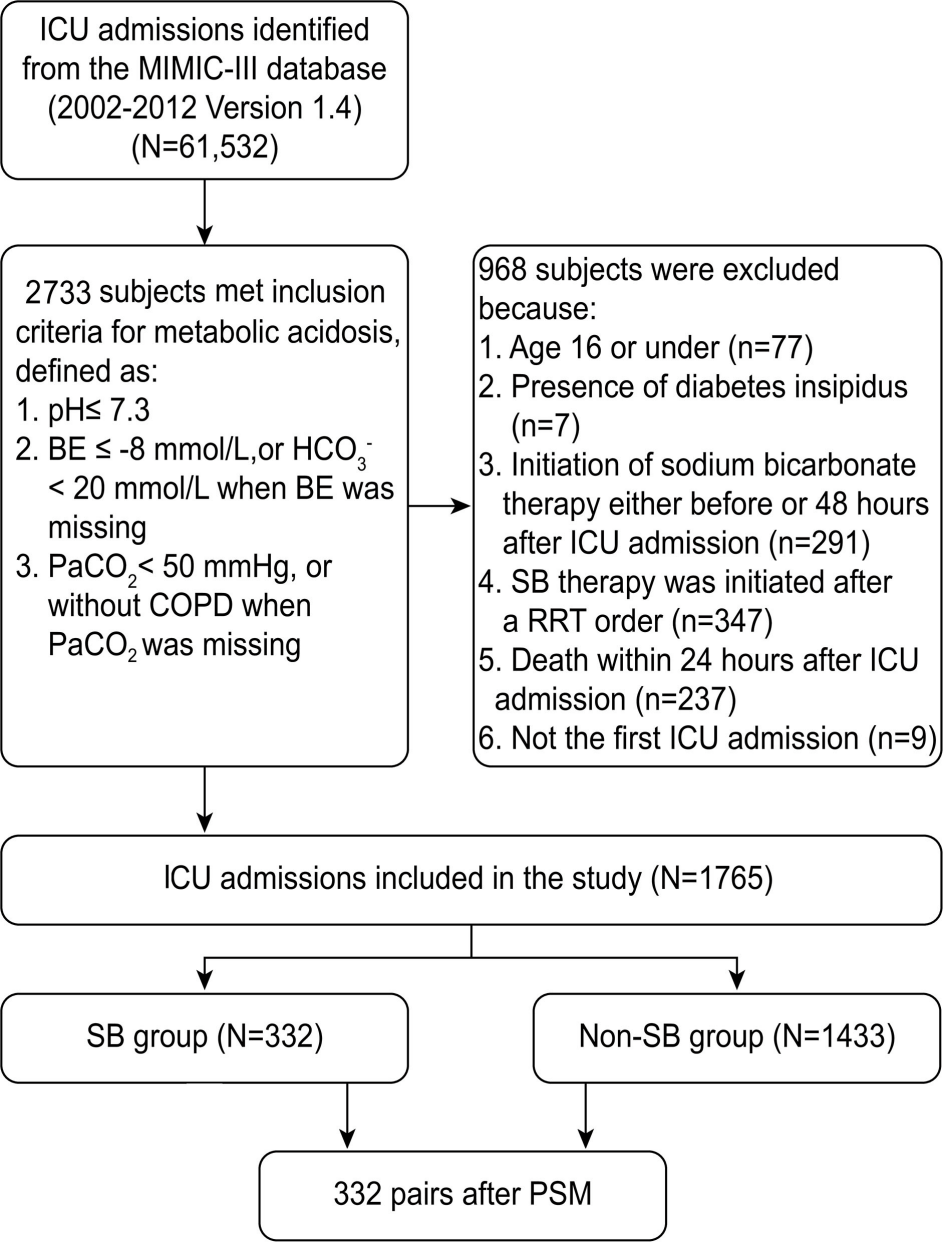
Flowchart of patient selection.

Out of 61,532 ICU records in the MIMIC-III database, 2,733 admissions fulfilled the definition of severe metabolic acidosis within 48 h after ICU admission. According to the inclusion and exclusion criteria, 1,765 patients were enrolled in this cohort. As shown in the flowchart of patient selection (Figure 1), 332 propensity score-matched pairs were generated and applied to the final analyses.

The baseline characteristics of patients in the SB group and non-SB group were compared before and after PSM. There were higher SOFA and SAPSII scores, a larger proportion of patients receiving mechanical ventilation and vasopressors, higher cumulative positive fluid balances at 24- and 48-h and more severe acidosis (i.e., blood gas analysis showed a significantly lower BE value, pH value, and AB value in the SB group than in the non-SB group before PSM) (Supplementary Table 2). Most of variables were comparable after PSM. Moreover, a significant difference between the two groups remained for variables including fluid balance at 24 and 48 h, SAPS II and SOFA scores, the proportion of patients with a diagnosis of sepsis receiving mechanical ventilation and vasopressors, and pH, PaCO_2_ and BE (Table 1).

### Association Between SB Therapy and Newly d/eOD

The median (IQR) volume of SB administered within 48 h after ICU admission was 60 (30, 150) ml (Table 1), which was initiated at 8.9 (4.45, 18.88) h after ICU admission, as shown in Supplementary Figure 2.

**Figure 2:**
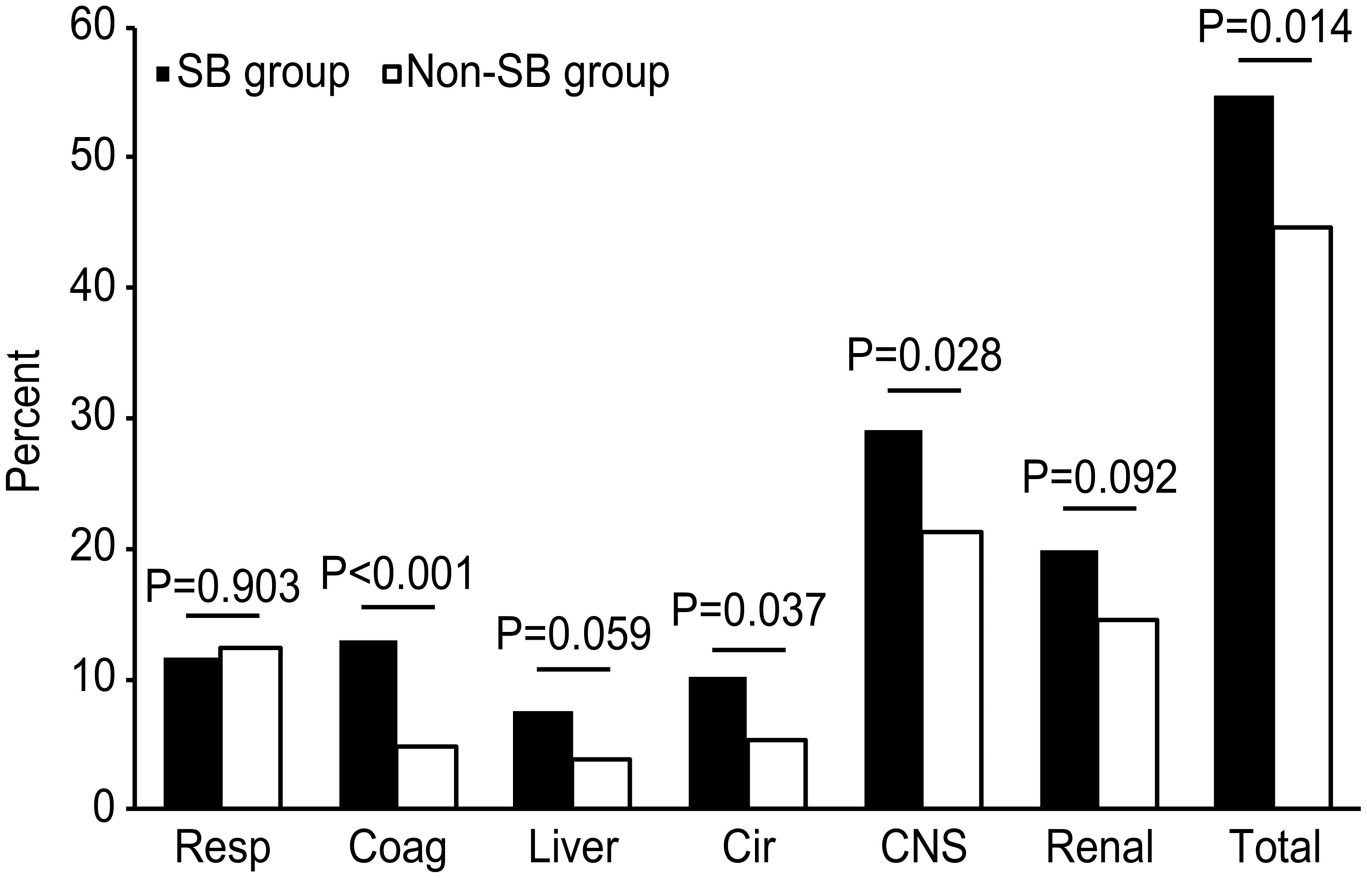
Comparison of the incidences of newly d/eOD between the SB and non-SB group after PSM. The chi-square test was used to compare the percentages of d/eOD between the two groups. d/eOD, developed or exacerbated organ dysfunction; composite of newly d/eOD was defined as an increase in SOFA score ≥2 over the baseline (SOFA score in the first 24 h) of one organ at least within 7 days of ICU admission. Resp, respiration; Coag, coagulation; Cir, circulation; CNS, central nervous system.

As shown in Figure 2, the incidence of newly d/eOD within 7 days of ICU admission was significantly increased in the SB group compared with the non-SB group (54.8 vs. 44.6%, *p* < 0.05), comprising central nervous system (CNS) dysfunction (29.1 vs. 21.2%, *p* < 0.05), coagulopathy (13.0 vs. 4.8%, *p* < 0.001) and circulation (10.1 vs. 5.4%, *p* < 0.05). A significant difference was not found in the incidence of respiratory, liver or renal dysfunction between the two groups.

**Figure 3:**
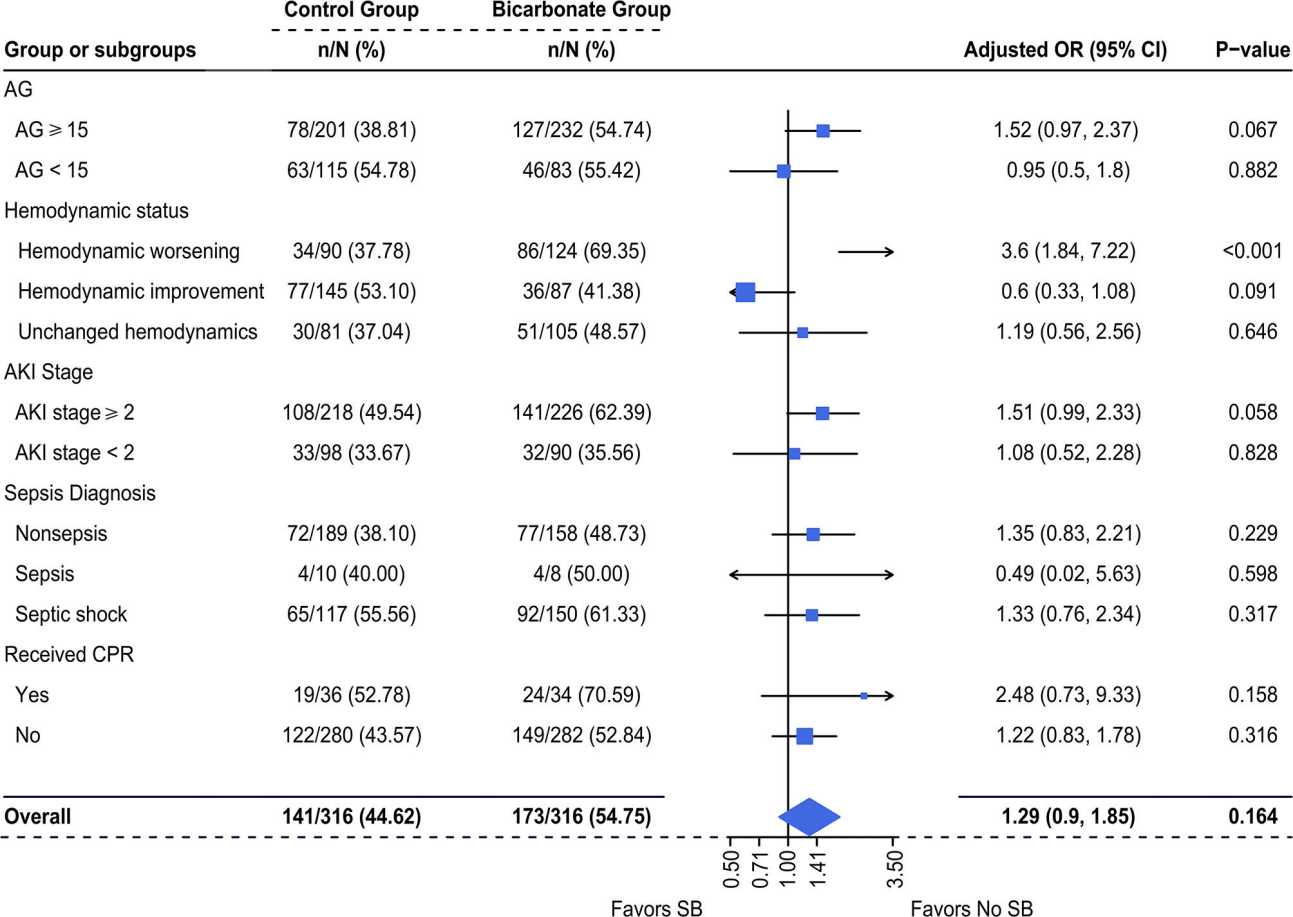
Multivariable-adjusted odds ratios of SBT for newly d/eOD. Multiple logistic regression was used. We adjusted for variables including (1) mechanical ventilation within 48 h after ICU admission, (2) sepsis diagnosis, (3) ketoacidosis diagnosis, (4) CPR, (5) cumulative fluid balance within 48 h after ICU admission, (6) SOFA score during the first 24 h after ICU admission and (7) pH-value. d/eOD, developed or exacerbated organ dysfunction; AKI, acute kidney injury; AG, anion gap; CPR, cardiopulmonary resuscitation.

Using univariable logistic regression, it was demonstrated that there was a composite of newly d/eOD within 7 days after ICU admission in 173/316 (54.8%) patients in the SB group vs. 141/316 (44.6%) patients in the non-SB group [OR (95% CI): 1.50 (1.10–2.06), *p* = 0.011, Supplementary Table 3]. Multivariable logistic regression revealed that the adjusted OR of SBT for this composite outcome was no longer significant [OR (95% CI): 1.39 (0.9, 1.85); *p* = 0.164, Figure 3]. The log-adjusted OR of SBT-associated newly d/eOD was also not significant in each quintile stratified by pH level (Supplementary Figure 3A).

The adjusted OR for risk potential of newly d/eOD subsequent to SBT was increased with a borderline significance in a subgroup of patients who had AG ≥ 15 [adjusted OR (95% CI): 1.52 (0.97, 2.37), *p* = 0.067] and AKI stage ≥2 [adjusted OR (95% CI): 1.51 (0.99, 2.33), *p* = 0.058, Figure 3]. This effect was neutral in the subgroup of patients with AG <15 [adjusted OR (95% CI): 0.95 (0.5, 1.8), *p* = 0.882] and AKI stage ≥2 [adjusted OR (95% CI): 1.08 (0.52, 2.28), *p* = 0.828, Figure 3]. It was significantly increased in the subgroup of patients with worsening hemodynamics [adjusted OR (95% CI): 3.6 (1.84, 7.22), *p* < 0.001], but not in the subgroup of patients with either improving hemodynamics or unchanged hemodynamics (Figure 3). In addition, there was no significant difference in the incidence of newly d/eOD between the SB group and the non-SB group among patients who were allocated into each sepsis subgroup, whether or not they received CPR (Figure 3).

### Impact of Hemodynamic Status on the Risk Potential of Newly d/eOD Subsequent to SBT

**Figure 4:**
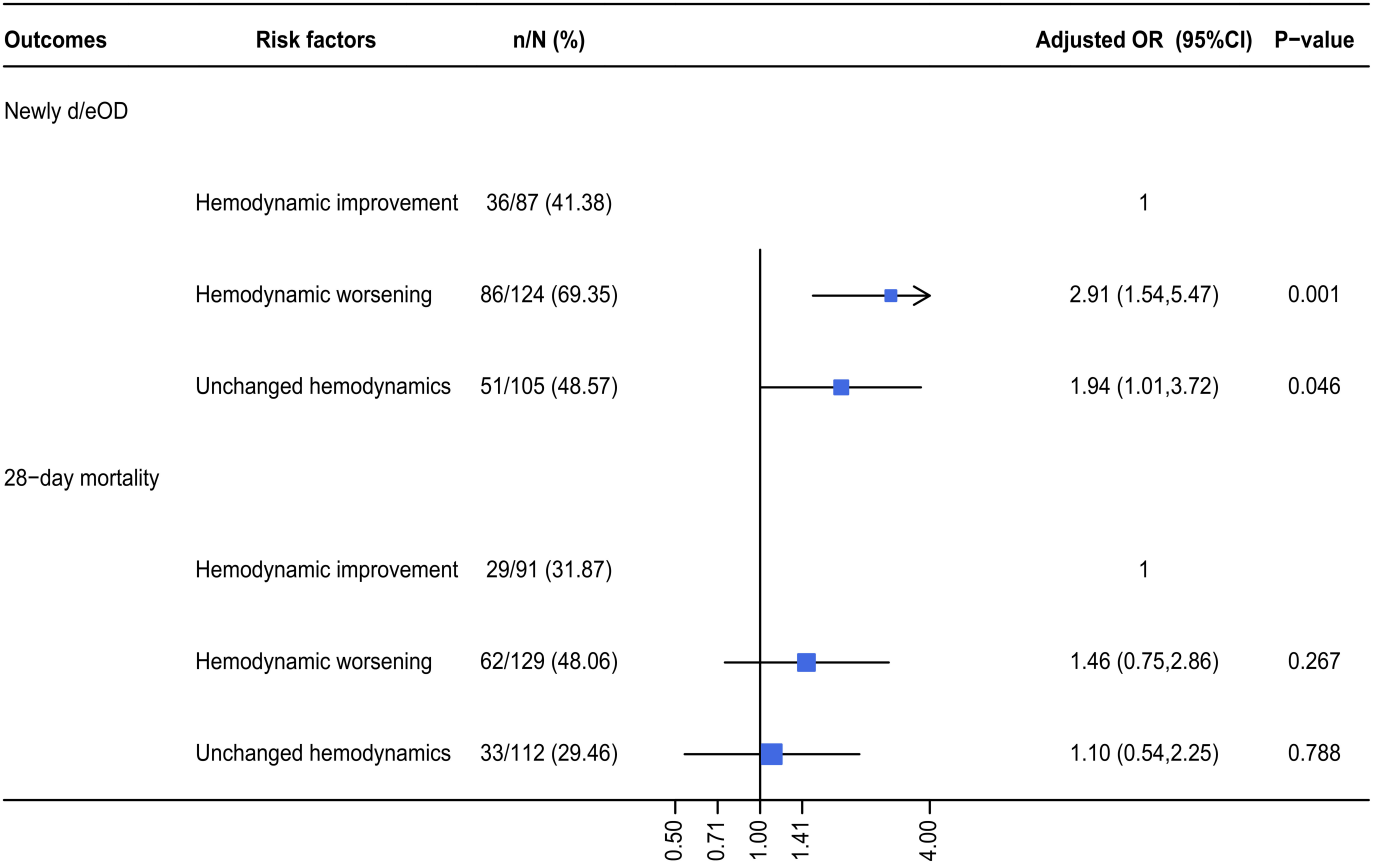
The adjusted OR for a composite of newly d/eOD and 28-day mortality in SB group. Multiple logistic regression was used. For the dependent variable of d/eOD and 28-day death, we adjusted variables including (1) mechanical ventilation within 48 h after ICU admission, (2) sepsis diagnosis, (3) ketoacidosis diagnosis, (4) CPR, (5) cumulative fluid balance within 48 h after ICU admission, (6) SOFA score during the first 24 h after ICU admission, and (7) pH-value. d/eOD, developed or exacerbated organ dysfunction.

The risk potential of newly d/eOD subsequent to SBT was further compared among patients who received SBT, but were characterized by different hemodynamic statuses. The subgroup of improving hemodynamics served as a reference for a multivariable logistic regression analysis. The results demonstrated that the risk potential of newly d/eOD subsequent to SBT within 7 days after ICU admission was significantly increased in the subgroup of patients characterized by both worsening hemodynamics [adjusted OR (95% CI): 2.91 (1.54, 5.47), *p* < 0.001] and unchanged hemodynamics [adjusted OR (95% CI): 1.94 (1.01, 3.72), *p* = 0.046, Figure 4).

### SBT-Associated 28-Day Mortality

**Figure 5:**
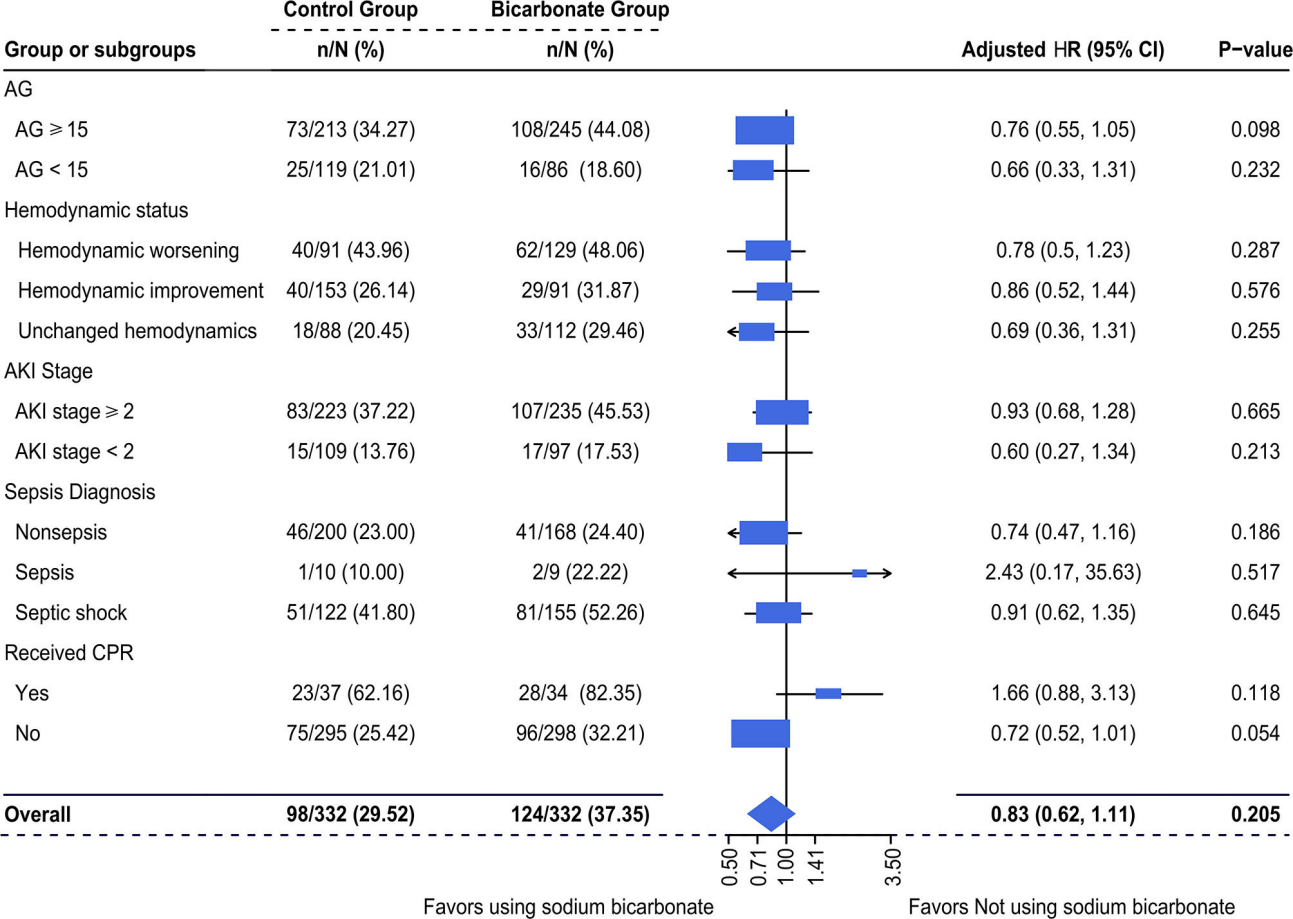
Multivariable-adjusted hazard ratios (HRs) of SBT for 28-day mortality. Multivariate Cox regression was used. We adjusted variables including (1) mechanical ventilation within 48 h after ICU admission, (2) sepsis diagnosis, (3) ketoacidosis diagnosis, (4) CPR, (5) cumulative fluid balance within 48 h after ICU admission, (6) SOFA score during the first 24 h after ICU admission, and (7) pH-value. d/eOD, developed or exacerbated organ dysfunction; AKI, acute kidney injury; AG, anion gap; CPR, cardiopulmonary resuscitation.

It was observed that there was an increased mortality in the SBT group compared with the control group (37.35 vs. 29.52%) in this study. However, the multivariable-adjusted hazard ratios (HRs) of 28-day mortality associated with SBT were no longer significant in acute critically ill patients who had metabolic acidosis [adjusted HR (95% CI): 0.83 (0.62–1.11), *p* = 0.205], as shown in Figure 5. Moreover, subgroup analysis failed to show a significantly increased hazard ratio (95% CI) of SBT for 28-day mortality. In addition, the log-adjusted OR of SBT for mortality was not significant in any quintile of patients stratified by pH (Supplementary Figure 3B).

## Discussion

In this study, it was observed that the incidence of a composite of newly d/eOD within 7 days of ICU admission was significantly higher in patients receiving SBT than in those not receiving SBT within 48 h of ICU admission who had metabolic acidosis (Figure 2). In addition, the adjusted odds ratio of this composite outcome subsequent to SBT was significantly increased in patients characterized by worsening hemodynamic status during initial resuscitation (within 6 h of ICU admission or before SBT if SB was given earlier), although it was not observed in the mixed overall patient population (Figure 3). Moreover, compared with patients who had improved hemodynamic status, patients with either worsening or unchanged hemodynamic status during initial resuscitation had a significantly higher risk potential for newly d/eOD subsequent to SBT (Figure 4). These findings suggested that patients could be at risk potential for development or exacerbation of organ dysfunction subsequent to SBT while their hemodynamic status remained unstable during the acute phase of critical illness.

Metabolic acidosis is common and associated with worse outcomes in critically ill patients (1, 27). Causal treatment is the best option but often differs according to the primary disease and is actually difficult in the acute phase of critical illness. Therefore, SBT is thought to counterbalance the negative effects of severe acidemia on organ function in clinical practice (8, 11). However, this approach has never been supported by prospective studies. For instance, two RCTs conducted by *Mathieu D* and *Cooper DJ* concluded that administration of sodium bicarbonate did not have a more favorable effect than saline solution on hemodynamics in critically ill patients who had lactic acidosis (14, 15). In this study, although hemodynamic changes immediately following SBT were not traced due to too many missing records in this database, it was found that the percentage of newly developed/exacerbated circulatory dysfunction (a net increase in SOFA score ≥2 in circulation within 7 days after admission) was markedly increased in the SBT group in comparison with the propensity score-matched control group (Figure 2). In addition, our results did not demonstrate a significantly beneficial effect of SBT on reducing the incidence of newly d/eOD in patients with metabolic acidosis, even in a subgroup of patients with improved hemodynamics during initial resuscitation (Figure 3). Moreover, the log of adjusted odds ratios of this composite outcome subsequent to SBT did not change significantly with any quintile of either pH (Supplementary Figure 3A). These results support the recommendations of the updated guidelines against using SB at pH > 7.15 in septic patients or 7.20 in intensive care patients complicated with moderate-to-severe acute renal insufficiency (19, 20). The rationale for SB infusion remains questionable for increasing clinical tolerance to severe acidemia or compensating for extra alkalinity loss beyond hemodynamic optimization in acute critically ill patients with metabolic acidosis.

Significantly, our study provided clinical evidence that SBT for metabolic acidosis raised the risk potential for subsequent organ dysfunction or exacerbation in acute critically ill patients who were characterized by an unstable hemodynamic status during initial resuscitation. Previously, the threat to organ function from the side effects of SBT was mainly based on the results of the potential for paradoxical intracellular acidosis in some *in vitro* experiments or animal studies (4, 28, 29). In theory, the administered bicarbonate reacted with acids to form water and CO_2_, which readily diffused across cell membranes. The high CO_2_ concentration could drive this same equation in reverse and generate intracellular H^+^. Additionally, administration of sodium bicarbonate significantly lowered plasma ionized calcium, which induced cell injury. In fact, the PaCO_2_ was found to increase by ~5 mmHg at 15 min following 2 mmol/kg bicarbonate infusion in patients with severe acidosis (14). However, no difference was detected with respect to organ dysfunction between the SBT group and the control group in these trials (14, 15), except for the data from animal studies (30). Although the percentage of patients with newly d/eOD was increased in the SBT group compared with the control group (54.8 vs. 44.6%), this side effect associated with SBT was neutral in all patients in this study (Figure 3). Interestingly, our results revealed that the adjusted odds ratio of SBT resulting in subsequent organ dysfunction was significantly increased in the subgroup of patients with worsening hemodynamics during initial resuscitation (Figure 3). In addition, this side effect was borderline significant in subgroups of patients with either AG values remaining ≥15 or severe AKI (fulfilling the KDIGO criteria for stage 2 and stage 3, Figure 3), who might benefit from further hemodynamic optimization in the acute phase of critical illness (31). Although the mechanism remains under investigation, our findings issued a warning against decision making for SBT before adequate hemodynamic optimization in acute critically ill patients with metabolic acidosis.

Similar to previous results (1, 9, 13, 16), our data suggested that SBT for metabolic acidosis within 48 h of ICU admission did not increase the adjusted odds ratio of 28-day mortality in critically ill patients (Figure 4). This impact of SBT was also not significant in any quintile stratified by plasma pH (Supplementary Figure 3B) and did not vary in patients with different hemodynamic statuses during initial resuscitation (Figure 5). However, it was observed that there was an increased mortality rate in the SBT group compared with the control group (37.35 vs. 29.52%) in this study. Although there were multiple factors, the limited number of recruited patients could be one of the reasons for these negative results. Further prospective research is needed to investigate the SBT-associated outcomes in critically ill patients with severe metabolic acidosis, especially for SBT given to patients with unstable hemodynamics during initial resuscitation.

There were several limitations in this study. First, this study was based on electronic healthcare records of routine clinical practice. Missing data was found for almost all variables. For instance, measurements regarding hemodynamic status, plasma electrolytes and blood gas analysis 1 h after initiating SBT were unavailable in most of the enrolled patients, which limited the mechanistic analysis of the effect of SB therapy for metabolic acidosis on organ dysfunction and mortality. Second, this is a retrospective study. The potential sources of bias might impair the reproducibility of the results. The two groups were different at the baseline in this cohort. These results indicated that the SBT group seemed to have a higher severity of disease, and to received SBT more probably. Actually, it is common in clinical practices for management of severe metabolic acidosis, especially for critically ill patients. For instance, Kim Hj reported that the severity of disease was significantly higher in group of patients treated with SB than that in group without use of SB in a retrospective cohort study (10). Owing to lack of solid data to guide severe acidosis management, SBT was therefore an alternative choice. Although we adjusted the potential confounders, we should interpret the results with caution. The association between SBT for metabolic acidosis and threatened organ function and mortality in patients who experienced hemodynamic worsening during initial resuscitation needs to be validated in a prospective trial. Third, the results of the relationship between pH and BE levels and organ function or mortality should be interpreted with caution owing to the small sample size. Thus, a well-designed prospective trial is needed to further investigate the place of SBT for metabolic acidosis in acute critically ill patients.

## Conclusions

Our findings did not demonstrate an association between SBT and outcomes in critically ill patients with metabolic acidosis. Compared with patients who had hemodynamic improvement, however, patients with either worsening or unchanged hemodynamic status during initial resuscitation had a significantly higher risk potential of newly d/eOD subsequent to SBT.

## Data Availability Statement

The raw data supporting the conclusions of this article will be made available by the authors, without undue reservation.

## Ethics Statement

The studies involving human participants were reviewed and approved by Peking University Third Hospital. Written informed consent for participation was not required for this study in accordance with the national legislation and the institutional requirements.

## Author Contributions

PM, TiaW, LY, and TieW designed the study protocol and interpreted the results. LZ, HZ, JX, and JH were responsible for data cleaning and statistical analysis. TieW and LY were the major contributors in writing the manuscript. ZZ helped to revise the manuscript. PM critically reviewed the manuscript and agreed with the final version and findings. All authors read and approved the submitted manuscript.

## Conflict of Interest

JH was employed by company Shanghai Palan DataRx Co. Ltd. The remaining authors declare that the research was conducted in the absence of any commercial or financial relationships that could be construed as a potential conflict of interest.

## Abbreviations

ICU: intensive care unit
SB: sodium bicarbonate
SBT: sodium bicarbonate therapy
d/eOD: newly developed or exacerbated organ dysfunction
AKI: acute kidney injury
MIMIC-III: Medical Information Mart for Intensive Care Database
RRT: renal replacement therapy
AG: anion gap
AB: actual bicarbonate
BE: base excess
Hct: hematocrit
Hb: hemoglobin
OR: odds ratio
aOR: adjusted odds ratio
HR: hazard ratio
aHR: adjusted hazard ratio
CPR: cardiac pulmonary resuscitation
SOFA: Sequential Organ Failure Assessment
MAP: mean arterial pressure
KDIGO: Kidney Disease, Improving Global Outcomes
SAPSII: The Simplified Acute Physiology Score II
PSM: Propensity score matching
CFB: cumulative fluid balance
MV: mechanical ventilation
CPR: cardiopulmonary resuscitation.
