# Supplementary material for: Risk Potential for Organ Dysfunction Associated With Sodium Bicarbonate Therapy in Critically Ill Patients With Hemodynamic Worsening
Source: Front Med (Lausanne). 2021 Jul 7;8:665907. doi: 10.3389/fmed.2021.665907 (PMC8292723; doi:10.3389/fmed.2021.665907)
Supplement: Supplementary file 1 [file Data_Sheet_1.docx]

**Supplement**

**Table S1**. Conversion to norepinephrine equivalents

| Drug | Dose | Norepinephrine equivalent | |
| --- | --- | --- | --- |
| Epinephrine | 0.1 μg/kg/min | | 0.1 μg/kg/min |
| Norepinephrine | 0.1 μg/kg/min | | 0.1 μg/kg/min |
| Dopamine | 15 μg/kg/min | | 0.1 μg/kg/min |
| Phenylephrine | 1 μg/kg/min | | 0.1 μg/kg/min |
| Vasopressin | 0.04 U/min | | 0.1 μg/kg/min |

**Table S2**. Characteristics of patients in the SB group and non-SB group before PSM

| **Characteristics** | **Before PSM** | | **P-value** |
| --- | --- | --- | --- |
|  | **SB group (N = 332)** | **Non-SB group (N = 433)** |  |
| **General characteristics** | | | |
| Age (year) | 66.5 (54.2, 77.6) | 66.0 (52.2, 78.4) | 0.673 |
| Sex (male, n (%)) | 158 (47.6%) | 748 (52.2%) | 0.146 |
| Category of disease (surgical, n (%)) | 88 (26.5%) | 527 (36.8%) | < 0.001 |
| With comorbidity, n (%) | 330 (99.4%) | 1375 (96.0%) | 0.003 |
| ICU admission via ED, n (%) | 204 (61.5%) | 705 (49.2%) | < 0.001 |
| Received CPR, n (%) | 34 (10.2%) | 106 (7.4%) | 0.106 |
| Sepsis, n (%) | 164 (49.4%) | 501 (35.0%) | < 0.001 |
| Septic shock, n (%) | 155 (46.7%) | 461 (32.2%) | < 0.001 |
| SAPS II score [M(IQR)] | 52 (40, 65) | 44 (34, 55) | < 0.001 |
| SOFA score [M(IQR)] | 8 (6, 12) | 6 (4, 9) | < 0.001 |
| **Organ dysfunction and supportive therapies within 48 hours of ICU admission** | | | |
| Mechanical ventilation, n (%) | 219 (66.0%) | 821 (57.3%) | 0.005 |
| Circulatory shock, n (%) | 285 (85.8%) | 1227 (85.6%) | 0.988 |
| Received vasopressors, n (%) | 227 (68.4%) | 855 (59.7%) | 0.004 |
| With AKI, n (%) | 269 (81.0%) | 1065 (74.3%) | 0.013 |
| AKI Stage = 1 | 34 (10.2%) | 237 (16.5%) |  |
| AKI Stage = 2 | 88 (26.5%) | 431 (30.1%) |  |
| AKI Stage = 3 | 147 (44.3%) | 397 (27.7%) |  |
| Received RRT, n (%) | 37 (11.14%) | 990 (69.1%) | < 0.001 |
| SB dosage [L, M(IQR)] | 60 (30, 150) | 0 | NA |
| **Laboratory tests within 48 hours of CU admission** | | | |
| pH | 7.21 (7.15, 7.25) | 7.24 (7.20, 7.27) | < 0.001 |
| PaCO_2_ [mmHg, M(IQR)] | 37.5 (31, 44) | 42 (37, 45) | < 0.001 |
| PaO_2_ [mmHg, M(IQR)] | 89 (61, 122) | 72 (48, 97) | < 0.001 |
| AB (mEq/L） | 15 (12, 17) | 17 (15, 19) | 0.020 |
| BE (mEq/L） | -12.5 (-16, -10) | -10 (-12, -9) | < 0.001 |
| Lactate (mmol/L) | 3.2 (1.7, 6.2) | 3.1 (1.8, 5.6) | 0.740 |
| Hct (%) | 27 (22, 32) | 28 (25, 32) | 0.038 |
| Hb (g/dL) | 9.0 (7.2, 10.7) | 9.3 (8.3, 10.6) | 0.047 |
| Lactate≥mmol/L, n (%) | 251 (75.6%) | 1025 (71.5%) | 0.154 |

Note: Baseline characteristic data were extracted within 6 hours of ICU admission or before SBT if it was initiated earlier. SB: Sodium bicarbonate; SBT: sodium bicarbonate therapy; SOFA: Sequential Organ Failure Assessment; MAP: mean arterial pressure; SAPSII: the Simplified Acute Physiology Score II; PSM: propensity score matching; AB: actual bicarbonate; BE: base excess; Hct: hematocrit; Hb: hemoglobin; AKI: acute kidney injury; RRT: renal replacement therapy; CFB: cumulative fluid balance; MV: mechanical ventilation; CPR: cardiopulmonary resuscitation.

**Table S3.** Association between SBT and a composite of newly d/eOD in patients with acidosis

| **Newly d/eOD** | **N** | **Univariable** | | **P-value** | **Multivariable** | | **P-value** |
| --- | --- | --- | --- | --- | --- | --- | --- |
|  |  | **OR** | **95% CI** |  | **adjusted OR** | **95% CI** |  |
| **Overall** | 632 | 1.5 | (1.1, 2.06) | 0.011 | 1.29 | (0.9, 1.85) | 0.164 |
| **AG** |  |  |  |  |  |  |  |
| AG ≥ 15 | 433 | 1.91 | (1.3, 2.81) | 0.001 | 1.52 | (0.97, 2.37) | 0.067 |
| AG < 15 | 198 | 1.03 | (0.58, 1.81) | 0.929 | 0.95 | (0.5, 1.8) | 0.882 |
| **Hemodynamic status*** |  |  |  |  |  |  |  |
| Hemodynamic worsening | 214 | 3.73 | (2.12, 6.67) | < 0.001 | 3.6 | (1.84, 7.22) | < 0.001 |
| Hemodynamic improvement | 232 | 0.62 | (0.36, 1.06) | 0.085 | 0.6 | (0.33, 1.08) | 0.091 |
| Unchanged hemodynamics | 186 | 1.61 | (0.89, 2.92) | 0.117 | 1.19 | (0.56, 2.56) | 0.646 |
| **AKI stage** |  |  |  |  |  |  |  |
| AKI stage≥2 | 444 | 1.69 | (1.16, 2.47) | 0.007 | 1.51 | (0.99, 2.33) | 0.058 |
| AKI stage < 2 | 188 | 1.09 | (0.59, 1.99) | 0.786 | 1.08 | (0.52, 2.28) | 0.828 |
| **Sepsis diagnosis** |  |  |  |  |  |  |  |
| Nonsepsis | 347 | 1.54 | (1.01, 2.38) | 0.047 | 1.35 | (0.83, 2.21) | 0.229 |
| Sepsis | 18 | 1.5 | (0.23, 10.41) | 0.672 | 0.49 | (0.02, 5.63) | 0.598 |
| Septic shock | 267 | 1.27 | (0.78, 2.08) | 0.342 | 1.33 | (0.76, 2.34) | 0.317 |
| **CPR** |  |  |  |  |  |  |  |
| With CPR | 70 | 2.15 | (0.81, 5.91) | 0.129 | 2.48 | (0.73, 9.33) | 0.158 |
| Without CPR | 562 | 1.45 | (1.04, 2.03) | 0.028 | 1.22 | (0.83, 1.78) | 0.316 |

**Note: Univariate analysis and** multiple logistic **regression were used. We adjusted variables including ① mechanical ventilation within 48 hours after ICU admission, ② sepsis diagnosis, ③ ketoacidosis diagnosis, ④ CPR, ⑤ cumulative fluid balance within 48 hours after ICU admission, ⑥ SOFA score during the first 24 hours after ICU admission and ⑦pH value.** SBT: Sodium bicarbonate therapy; **d/eOD: developed or exacerbated organ dysfunction; AKI: acute kidney injury; AG: anion gap; CPR: cardiopulmonary resuscitation.**

**Table S4.** Association between SBT and 28-day mortality in patients with acidosis

|  | **N** | **Univariable** | | **P-value** | **Multivariable** | | **P-value** |
| --- | --- | --- | --- | --- | --- | --- | --- |
|  |  | **HR** | **95% CI** |  | **adjusted HR** | **95% CI** |  |
| **Overall** | 664 | 1.3 | (1, 1.7) | 0.05 | 0.83 | (0.62, 1.11) | 0.205 |
| **AG** |  |  |  |  |  |  |  |
| AG ≥ 15 | 458 | 1.32 | (0.98, 1.78) | 0.065 | 0.76 | (0.55, 1.05) | 0.098 |
| AG < 15 | 205 | 0.88 | (0.47, 1.65) | 0.688 | 0.66 | (0.33, 1.31) | 0.232 |
| **Patients subgrouped with** |  |  |  |  |  |  |  |
| **Hemodynamic Improvement** |  |  |  |  |  |  |  |
| Hemodynamic worsening | 220 | 1.08 | (0.72, 1.6) | 0.72 | 0.78 | (0.5, 1.23) | 0.287 |
| Hemodynamic improvement | 244 | 1.23 | (0.76, 1.98) | 0.396 | 0.86 | (0.52, 1.44) | 0.576 |
| Unchanged hemodynamics | 200 | 1.46 | (0.82, 2.6) | 0.196 | 0.69 | (0.36, 1.31) | 0.255 |
| **AKI STAGE** |  |  |  |  |  |  |  |
| AKI stage ≥ 2 | 458 | 1.26 | (0.94, 1.67) | 0.12 | 0.93 | (0.68, 1.28) | 0.665 |
| AKI stage < 2 | 206 | 1.3 | (0.65, 2.6) | 0.46 | 0.6 | (0.27, 1.34) | 0.213 |
| **SEPSIS OR SHOCK STATUS** |  |  |  |  |  |  |  |
| Nonsepsis | 368 | 1.07 | (0.7, 1.62) | 0.767 | 0.74 | (0.47, 1.16) | 0.186 |
| Sepsis | 19 | 2.32 | (0.21, 25.63) | 0.492 | 2.43 | (0.17, 35.63) | 0.517 |
| Septic shock | 277 | 1.29 | (0.91, 1.84) | 0.149 | 0.91 | (0.62, 1.35) | 0.645 |
| **CPR** |  |  |  |  |  |  |  |
| With CPR | 71 | 1.63 | (0.94, 2.83) | 0.085 | 1.66 | (0.88, 3.13) | 0.118 |
| Without CPR | 593 | 1.29 | (0.95, 1.75) | 0.097 | 0.72 | (0.52, 1.01) | 0.054 |

**Note: Univariate analysis and multivariate Cox regression were used. We adjusted variables including ① mechanical ventilation within 48 hours after ICU admission, ② sepsis diagnosis, ③ ketoacidosis diagnosis, ④ CPR, ⑤ cumulative fluid balance within 48 hours after ICU admission, ⑥ SOFA score during the first 24 hours after ICU admission and ⑦ pH value.** SBT: Sodium bicarbonate therapy; **d/eOD: developed or exacerbated organ dysfunction; AKI: acute kidney injury; AG: anion gap; CPR: cardiopulmonary resuscitation.**

Index

Parameter

Category

Norepinephrine

Norepinephrine decline>0.1μg/min/kg or the rate of decline>20%

Hemodynamic improving

Norepinephrine ascending >0.1μg/min/kg or the rate of ascending>20%

Hemodynamic worsening

Norepinephrine decline or ascending≤0.1μg/min/kg, the rate of decline≤20% and the rate of ascending≤20%

lactate

lactate>2mmol/L drop to <2mmol/L or lactate clearance>10%

乳酸清除率>10%

lactate<2mmol/L ascend to >2mmol/L or lactate ascending>10%

Lactate always <2mmol/L , lactate clearance<10% and lactate ascending<10%

Hemodynamic unchanging

**Figure S1.** Classification of hemodynamic status

**Figure S2.** Time distribution of SB therapy initiation within 48 hours of ICU admission.

Legend: The start time of sodium bicarbonate treatment was nonnormally distributed. Sodium bicarbonate was infused within 12 hours after ICU admission in most patients, and the median was 8.9 (4.45, 18.88) hours.


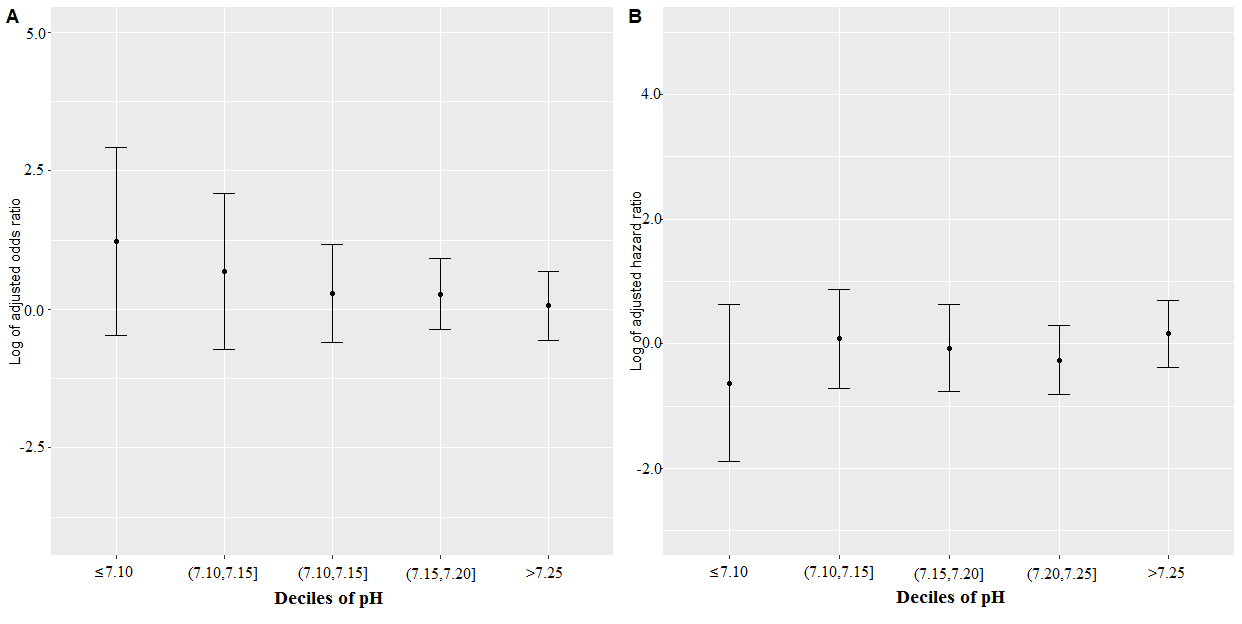


**Figure S3**. Log of adjusted hazard ratios of SBT-associated newly d/eOD and 28-day mortality in patients stratified by pH.

Legend: Multiple logistic regression was used. Stratification was based on the lowest pH value before SBT. The X-axis shows quintiles of pH. The log-adjusted odds ratios of SBT-associated newly d/eOD (S2A) and 28-day mortality (S2B) were not significant in patients in any quintile of pH values. d/eOD: developed or exacerbated organ dysfunction.
